# Performance of GPS/GPRS tracking devices improves with increased fix interval and is not affected by animal deployment

**DOI:** 10.1371/journal.pone.0265541

**Published:** 2022-03-30

**Authors:** Marta Acácio, Philip W. Atkinson, João Paulo Silva, Aldina M. A. Franco

**Affiliations:** 1 School of Environmental Sciences, University of East Anglia, Norwich, United Kingdom; 2 British Trust for Ornithology, The Nunnery, Thetford, United Kingdom; 3 CIBIO/InBio, Centro de Investigação em Biodiversidade e Recursos Genéticos, Laboratório Associado, Universidade do Porto, Vairão, Portugal; 4 CIBIO/InBio, Centro de Investigação em Biodiversidade e Recursos Genéticos, Laboratório Associado, Instituto Superior de Agronomia, Universidade de Lisboa, Lisboa, Portugal; 5 BIOPOLIS Program in Genomics, Biodiversity and Land Planning, Universidade do Porto, Vairão, Portugal; University of Bucharest, ROMANIA

## Abstract

The use of GPS tracking technologies has revolutionized the study of animal movement providing unprecedentedly detailed information. The characterization of GPS accuracy and precision under different conditions is essential to correctly identify the spatial and temporal resolution at which studies can be conducted. Here, we examined the influence of fix acquisition interval and device deployment on the performance of a new GPS/GSM solar powered device. Horizontal and vertical accuracy and precision of locations were obtained under different GPS fix acquisition intervals (1min, 20 min and 60 min) in a stationary test. The test devices were deployed on pre-fledgling white storks (*Ciconia ciconia*) and we quantified accuracy and precision after deployment while controlling for bias caused by variation in habitat, topography, and animal movement. We also assessed the performance of *GPS-Error*, a metric provided by the device, at identifying inaccurate locations (> 10 m). Average horizontal accuracy varied between 3.4 to 6.5 m, and vertical accuracy varied between 4.9 to 9.7 m, in high (1 min) and low frequency (60 min) GPS fix intervals. These values were similar after the deployment on white storks. Over 84% of GPS horizontal positions and 71% of vertical positions had less than 10m error in accuracy. Removing 3% of data with highest *GPS-Error* eliminated over 99% of inaccurate positions in high GPS frequency intervals, but this metric was not effective in the low frequency intervals. We confirmed the suitability of these devices for studies requiring horizontal and vertical accuracies of 5-10m. For higher accuracy data, intensive GPS fix intervals should be used, but this requires more sophisticated battery management, or larger batteries and devices.

## Introduction

The collection of animal movement data has substantially benefited from rapid technological advances. New tracking technologies enable researchers to unravel novel patterns of animal behavior and collect detailed spatial and temporal resolution data that can inform species conservation and management [1]. High accuracy Global Positioning System (GPS) technology devices are now commonly used to track wildlife across taxa and environments and have improved the study of animal movement and habitat use [2, 3], revolutionizing the field of movement ecology [4].

The GPS location data can be archived or remotely transmitted by the tracking devices. The ability to remotely transmit data is particularly convenient and allows researchers to track animals that are difficult to recapture and to collect data at higher fix intervals, since data is recovered regardless of the animals’ movements or the device memory [3]. There are several transmission protocols, but Global System for Mobile communications / General Packet Radio Service (GSM/GPRS) has become widely used enabling worldwide transmission of large quantities of GPS data with reduced communications costs [1]. The combination of affordable remote transmission of data with solar power energy led to an improvement in device longevity and an exponential increase in tracking data collection [3].

The spatial resolution of the location data obtained by GPS tracking devices can vary due to environmental and technical reasons. The main environmental sources of variability in GPS device performance are topography [5, 6] and habitat [7–10]. GPS accuracy tends to decrease in areas with closed canopy forests [11–17] (but see [18]), where GPS fix acquisition rates are reduced given the attenuation of GPS signal resulting from poor sky availability [6, 7, 17, 19]. Moreover, in solar powered devices, fix acquisition rates can be reduced due to poor charging conditions [17]. Accuracy variability can also result from technical aspects related with signal acquisition, for example, the number [20] and geometry of satellites in the sky [19], which is quantified by the dilution of precision (DOP) metric. Higher DOP values indicate lower accuracy and can be due to poor satellite configuration, low number of satellites available, or increased triangulation errors due to clustering of satellites [16, 19, 20]. Both the number of satellites [5, 13, 21] and DOP [13, 18, 19, 22] are metrics that can be used to identify and eliminate low accuracy locations, but these methods tend to be poor predictors of fix accuracy [7, 17, 23].

After attaching the GPS device to the animal (hereafter, device deployment), the morphology, movement and behavior of animals can also influence both accuracy [7, 24] and fix acquisition success [13, 25–27] (but see [28]), hence device accuracy should be quantified before and after deployment. Stationary tests can be used to quantify performance before deployment, by comparing the distance between the estimated location given by the tracking device and the true location obtained by an independent method. These tests can provide realistic assessments of location error and determine device accuracy [13, 17, 22, 23]. However, it is difficult to assess device performance after deployment, as it requires knowing the exact positions of the animals after deployment [28, 29], thus accuracy after deployment is normally assessed using pets [7, 10].

It is important to quantify the spatial resolution of data obtained from tracking devices and provide accuracy estimates to the locations used in research applied to conservation and policy making [1]. Low horizontal GPS accuracy can detrimentally affect habitat selection studies, leading to poor model precision [30–33], while low vertical accuracy can be critical when determining flight altitude [34, 35], collision risk with human infrastructures [36–38], and for determining 3D habitat utilization distributions of airborne animals [37, 39]. Determining ways to identify low accuracy positions would enable researchers to increase the quality of the location datasets obtained and minimize the constraints caused by low accuracy GPS locations.

With an increasing use of GPS tracking technology, new devices are currently being designed and developed. Differences in hardware and software can influence the performance of tracking devices [6, 15, 30, 35], therefore it is critical to assess their accuracy and precision in order to understand their applicability in ecological studies. Here, we describe a novel GPS/GPRS wildlife tracking device and quantify its horizontal and vertical accuracy and precision in stationary tests and after deployment on large birds. We examine device variability and assess if GPS-Error, a metric calculated by the GPS device, can be used to identify low-accuracy locations. We assess the performance of the devices in field conditions and discuss their use in ecological and conservation studies.

## Materials and methods

### GPS/GPRS devices

The Movetech Telemetry Flyways-50 is a compact Quad-band GPS/GPRS unit, a 22% efficiency solar cell, a Lithium-Ion battery, and a nylon plastic 3-D printed housing. The device weight starts at 23g. In this test we used the 50g model suitable for deployment on large birds, such as white storks [40–42] or Spanish imperial eagles [43]. The GPS/GPRS unit contains a GPS module with an on-board chip antenna. The GPS determines the 3D fix coordinates (horizontal and altitude above the ellipsoid) when 4 or more satellites are in view. The device can be programmed to log GPS data from 1 second to 24 hours, allowing for different day and night intervals. The intervals can be updated over the air, permitting an adaptation of the schedule to new environmental conditions.

The GPS unit provides an estimate of the positional error (hereafter *GPS-Error*), which considers the maximum latitude/longitude position displacement in meters with a probability of 67% (i.e., ± 1 standard deviation). This metric is calculated directly by the GPS module and is more reliable than using single metrics of error (e.g. Horizontal Dilution of Precision or number of satellites used to obtain the fix) [44]. The data can be transferred to Movebank [45], and then visualized and downloaded for further analysis.

The GSM/GPRS unit, coupled with an agnostic SIM-card, provides global cellular connectivity and there is no external antenna, minimizing drag and interference with animal movements. These are archival devices with a memory for over 60,000 records, reducing the risk of data loss when the animal is in areas without GPRS network.

### Accuracy and precision in stationary test

We assessed the accuracy (closeness of the GPS locations to a known location, in meters) and precision (closeness of the GPS locations to each other, in meters) of the devices in a stationary test, using 11 GPS/GPRS tracking devices, fully sealed within a nylon plastic housing of medium thickness (between 1.5–2 mm) and ready for animal deployment. The stationary test was completed on a triangulation station located in Southern Portugal. The surrounding landscape is characterized by low altitude, slightly undulating plains, with large areas of non-irrigated agricultural land and low density of cork (*Quercus suber*) and holm-oak trees (*Quercus ilex*). We placed all the devices on top of a triangulation station simultaneously, at about 2 m above the ground, with a clear and uninterrupted view of the sky and programmed the devices over the air at three fix intervals: collecting GPS data every 1 min, 20 min and 60 min.

Horizontal accuracy was calculated as the distance between the coordinates obtained by the devices and the precise coordinates of the triangulation station, provided by the Direção-Geral do Território (DGT) [46]. Vertical accuracy was calculated as the difference between the altitude above the ellipsoid of the top of the triangulation station, and the altitude obtained by the devices. Negative vertical accuracy values are obtained when the GPS device’s altitude value is higher than the true altitude, and positive values result from a reading smaller than the true altitude. Hence, we quantified biases in under or overestimation of vertical locations.

The horizontal precision was determined using the mean and standard deviation of the geodesic distance between all locations obtained by the tracking devices, and the vertical precision as the mean absolute difference between all altitude readings of each device.

We performed a Kruskal-Wallis statistical analysis to assess differences in accuracy and precision between devices. We used data from all devices to compare the accuracy and precision of the positions collected at different fix intervals.

### Identification of inaccurate positions

We examined if the *GPS-Error* metric, calculated by the device, could be used to identify horizontal and vertically inaccurate positions (horizontal and vertical locations with more than 10 m error). Location error was classified in three categories: 11–20 m, 21–30 m and larger than 30 m. For each tracking device, we excluded 1%, 3%, 5% and 10% of the positions with highest *GPS-Error* and determined the proportion of locations with above errors remaining in the dataset. We compared the reliability *of GPS-Error* at identifying the locations with the highest vertical and horizontal error for the three device schedules tested.

### Accuracy and precision after deployment on birds

To assess the accuracy and precision of GPS devices before deployment, we performed a stationary test on 17 GPS-GPRS devices, programmed with 20 min fix interval, fully sealed within a reinforced housing (between 3–4 mm thickness) and ready for deployment on white storks. The GPS devices were left in the triangulation station for a minimum of 4 days and a maximum of 15 days. We calculated the horizontal and vertical position of the triangulation station by averaging 3 GPS positions and 3 altitude readings collected with a Ashtech ProMark 220 and an Ashtech 660 external antenna, on differential GPS mode (dGPS). The dGPS provided readings with a horizontal accuracy of 0.98 m (± 0.07 m) and vertical accuracy of 0.57 m (±0.42 m). By using the dGPS coordinates instead of the coordinates provided by DGT, we were able to replicate this protocol to calculate the precise location of the white stork nests and reliably compare the performance of the GPS devices before and after deployment.

After the stationary test, the 17 devices were deployed on white stork pre-fledging chicks (*Ciconia ciconia*) on nests located in the same region as the stationary test (approximately 50 km radius), in order to control for possible GPS sources error, such as different topography or habitat. The white stork chicks tagged were approximately 50 days old, had a minimum wing length of 400 mm and minimum weight 2.9 kg. The device and harness weighted less than 3% of the storks’ body weight. The loggers were back-mounted using a Teflon harness, with a weak link consisting of biodegradable cotton stitches below the sternum [40, 42]. This study was carried out in accordance with the recommendations of Instituto da Conservação da Natureza e das Florestas and was approved by the Animal Welfare & Ethical Review Board from the School of Biological Sciences at the University of East Anglia. Licenses to deploy the loggers were granted by the Instituto da Conservação da Natureza e das Florestas (license number 364/2020/CAPT to 368/2020/CAPT).

The nests were located on top of trees providing the devices a clear and uninterrupted view of the sky. To calculate the precise horizontal and vertical position of the nest, we averaged 3 GPS coordinates collected with the dGPS on top of the nest. To calculate the tracking device accuracy and precision after deployment we considered the GPS positions collected during the first 7 days after deployment to guarantee the data was obtained prior to fledgling, as white stork juveniles do not fledge before 65 days. Horizontal and vertical accuracy, and horizontal and vertical precision of the devices before and after deployment were calculated as described above. We performed a Kruskal-Wallis statistical analysis to assess differences in accuracy and precision before and after deployment. All analysis were performed in R software [47], and distances calculated using package *geosphere* [48].

## Results

### Stationary test

During the stationary test, we collected a variable number of GPS fixes per device using three GPS fix collection intervals (1 min, 20 min and 60 min), with a 100% fix acquisition rate (Table 1).

**Table 1:** Number of devices and locations collected during stationary and deployment tests.

Treatment	Fix interval	Number of devices	Number of locations	Horizontal		Vertical	
				Accuracy Mean (sd)	Precision Mean (sd)	Accuracy Mean (sd)	Precision Mean (sd)
**Stationary**	**1 min**	1	1929	3.40 (3.10)	4.93 (4.15)	4.95 (4.12)	3.60 (5.94)
	**20 min**	10	2203	4.23 (4.28)	6.14 (5.46)	6.56 (6.72)	8.79 (9.17)
	**60 min**	7	1488	6.50 (8.34)	9.15 (9.46)	9.69 (19.28)	14.31 (24.95)
**Before Deployment**	**20 min**	17	7333	4.21 (18.0)	7.10 (23.4)	7.00 (71.0)	11.00 (85.1)
**After Deployment**	**20 min**	17	5204	4.10 (15.0)	6.72 (19.7)	6.00 (56.0)	10.00 (66.8)

Mean and standard deviation of horizontal and vertical accuracy and precision, in meters, in the different fix intervals.

**Fig 1:**
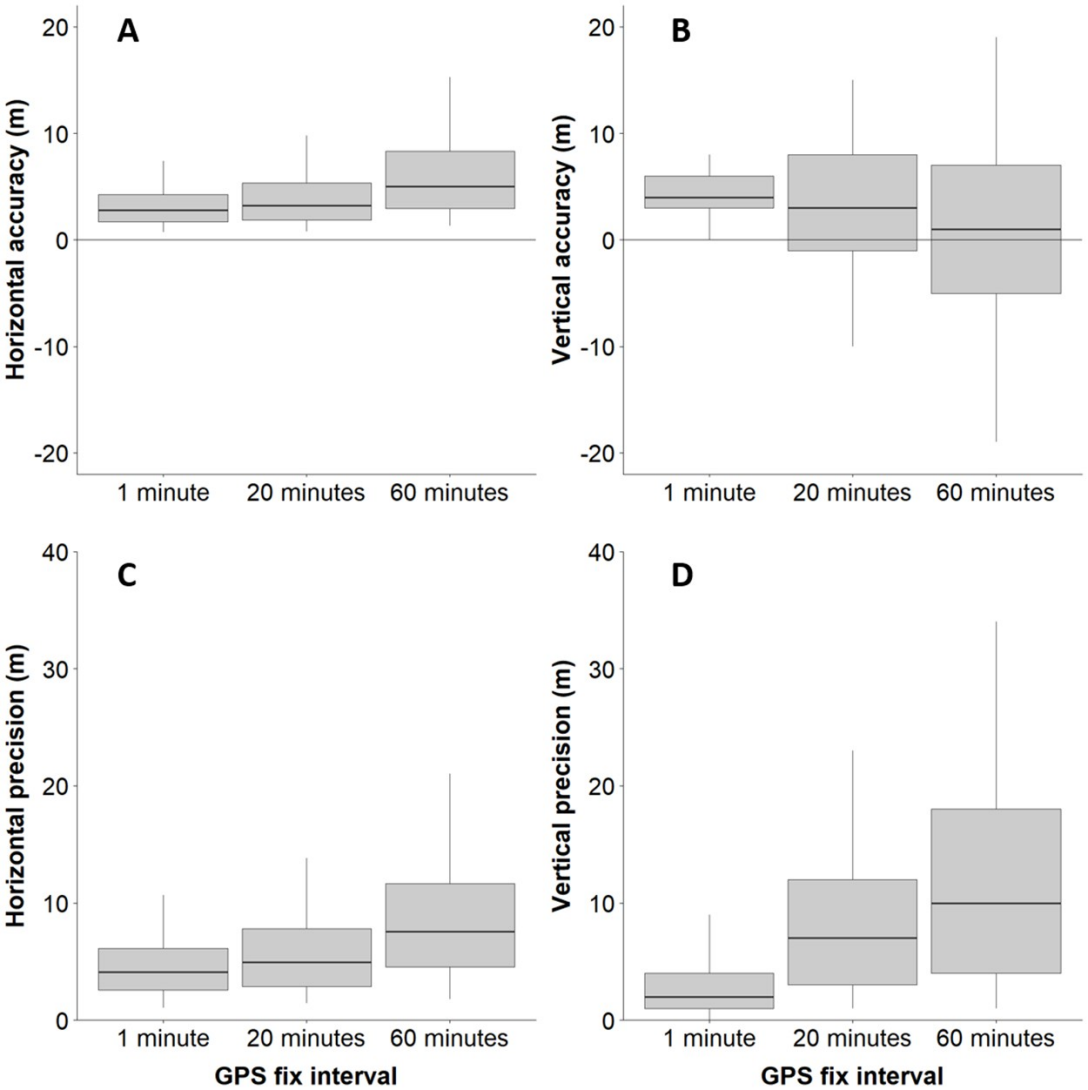
Horizontal and vertical accuracy and precision of devices programmed in different GPS fix intervals. Horizontal (A) and vertical (B) accuracy, and horizontal (C) and vertical (D) precision in meters of devices programmed with fix intervals of 1 minute, 20 minutes and 60 minutes. The box represents 25, 50 and 75% of the data and the error bar represents 5% and 95% of the data.

There was a significant decrease in horizontal (χ2 = 508.07, df = 2, p-value <0.001) and vertical (χ2 = 168.23, df = 2, p-value <0.001) accuracy and horizontal (χ2 = 108.41, df = 2, p-value <0.001) and vertical (χ2 = 361.90, df = 2, p-value <0.001) precision with increasing GPS fix acquisition intervals (Fig 1). The horizontal accuracy in the 1 min fix interval was 3.40 m (±3.10 m) and vertical accuracy was 4.95 m (±4.12 m) and decreased to 6.50 m (±8.34 m) and 9.69 m (±19.28 m) horizontal and vertical accuracy, respectively, in the 20 and 60 min fix interval. Vertical location error was approximately symmetric around zero during the longer fix intervals; during short intervals, the vertical errors were always positive, indicating a consistent underestimation of true altitude. Precision was also influenced by the fix collection interval. In the 1 min interval, the horizontal precision was 4.93 m (± 4.15 m) and vertical precision 3.60 m (± 5.90 m). In the 60 min interval, the horizontal precision was 9.15 m (± 9.46 m) and vertical precision 14.31 m (± 24.95 m), with intermediate values in the 20 min interval (horizontal precision = 6.14 m ± 5.46 m, vertical precision = 8.79 m ± 9.17 m) (Table 1).

**Fig 2:**
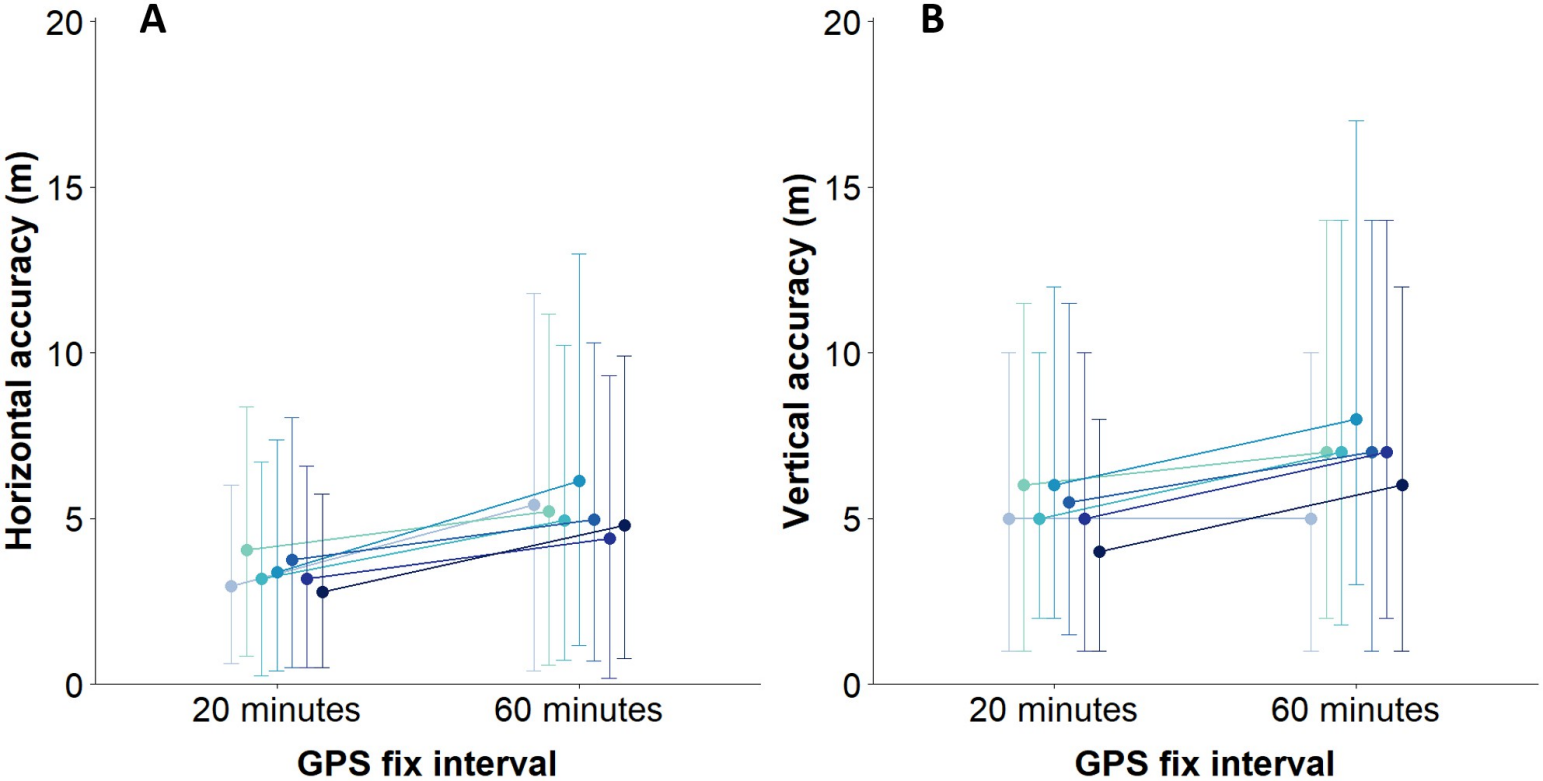
Horizontal and vertical accuracy variability of devices programmed on a 20 and 60 min GPS fix interval. Horizontal (A) and vertical (B) accuracy of tracking devices programmed to collect GPS locations every 20 and 60 minutes. The error bars represent 95% confidence intervals.

There was significant variability in accuracy between devices both in the 20 min (χ2 = 82.46, df = 9, p-value <0.001) and 60 min interval (χ2 = 22.62, df = 6, p-value <0.001), however, all devices consistently increased in accuracy in higher frequency GPS fix collection intervals (Fig 2).

### Identification of inaccurate positions

**Fig 3:**
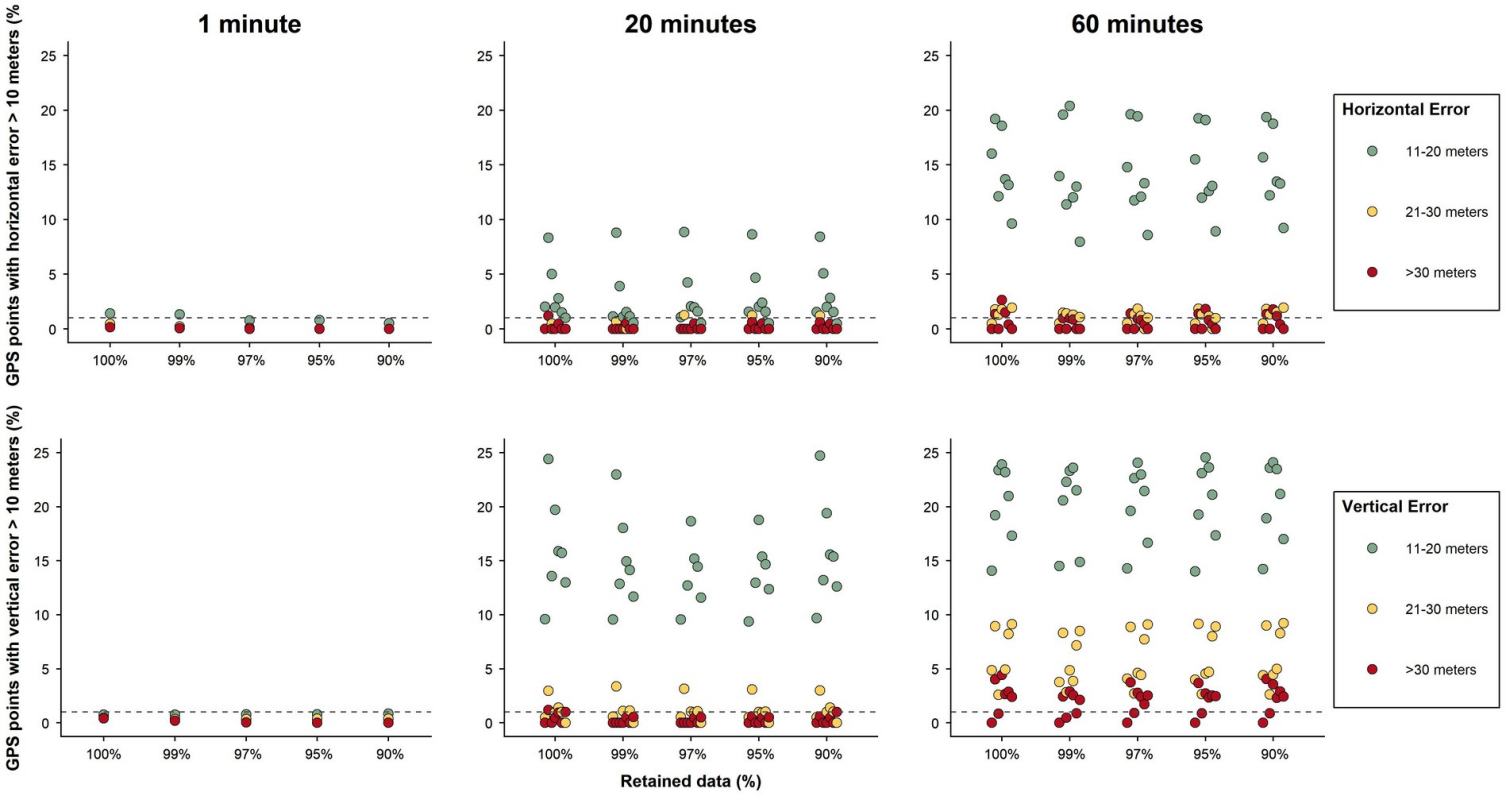
Identification of inaccurate positions in different GPS fix intervals. Percentage of locations with horizontal and vertical error larger than 10 meters for each device, and after removing the points with largest *GPS-Error* and remaining with 99%, 97%, 93%, 95% and 90% of the original data. The dash line indicates 1% of locations with vertical and horizontal errors larger than 10 meters.

The proportion of horizontally accurate positions (location error < 10 m) varied according to the device (Fig 3). Approximately 98% and 96% of the positions had a horizontal error below 10 m in the high frequency 1 min and 20 min intervals, respectively. The proportion of accurate locations declined to 83%, in the 60 min fix interval. Vertical accuracy also declined from 98% in the high frequency interval to approximately 71% in the 60 min fix intervals. *GPS-Error* provided a good metric to identify the locations that were less accurate in high intensity fix intervals. Eliminating 3% of the data with the highest *GPS-Error* obtained with the 1min fix interval, reduced 99% of positions with ≥10 m horizontal and vertical errors. *GPS-Error* was not effective at identifying the inaccurate locations in the less intensive schedules (Fig 3).

### Performance after deployment

**Fig 4:**
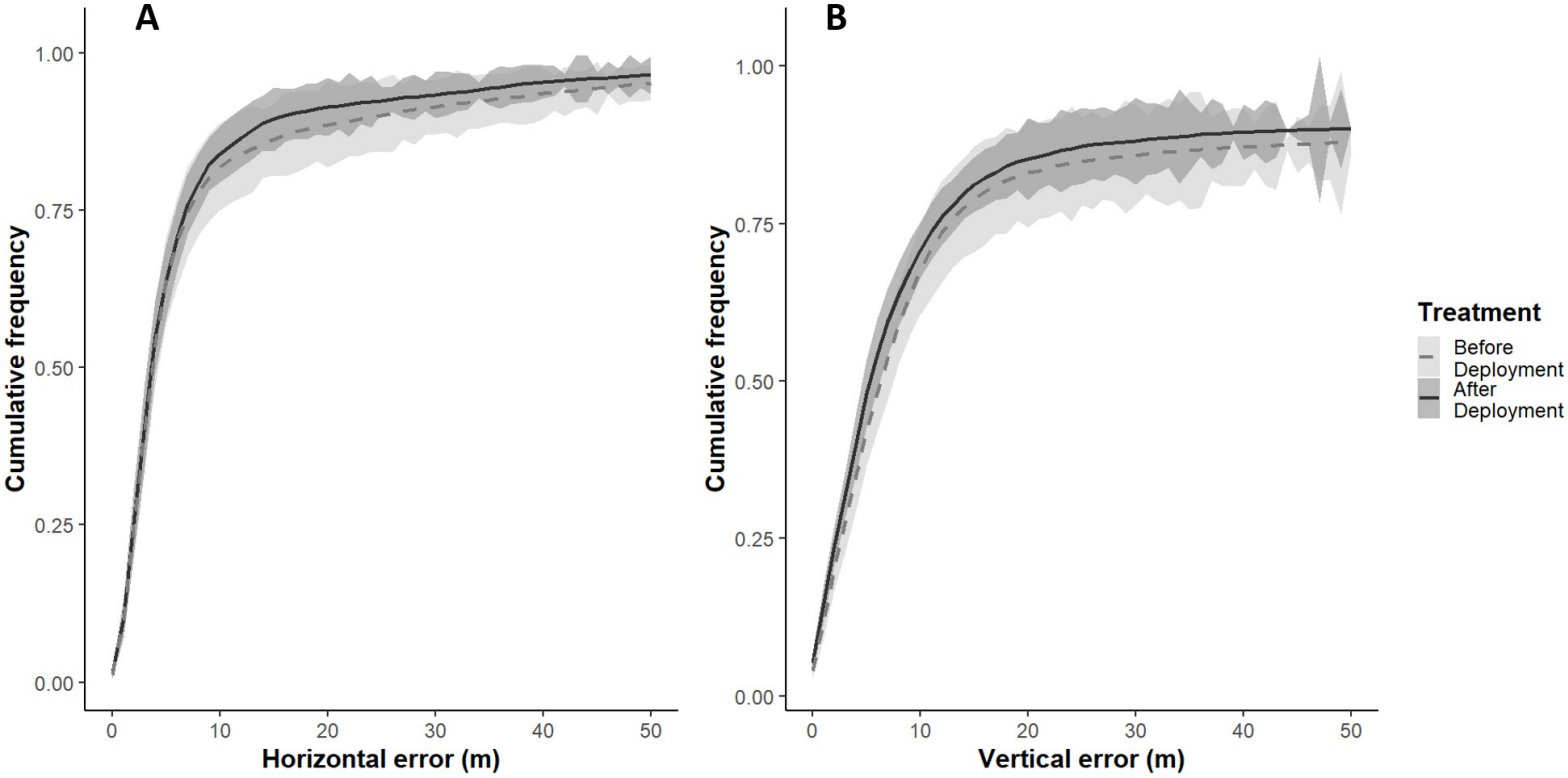
Cumulative frequency of horizontal and vertical accuracy before and after deployment on white storks. Cumulative frequency of (A) horizontal and (B) vertical errors (in m) of 16 GPS/GPRS devices before deployment (grey dashed line) and after deployment on white storks (black line). Shaded areas represent the standard deviation of the errors.

In total, across all 16 devices we collected 7,333 positions during the stationary test and 5,204 GPS positions during the deployment test. Horizontal accuracy did not change after deployment of the devices on white storks (χ2 = 3.80, df = 1, p-value = 0.051), the mean accuracy was 4.21 m (± 18 m) before and 4.10 m (± 15 m) after deployment (Fig 4). Vertical accuracy improved after deployment (χ2 = 43.72, df = 1, p-value <0.001), from 7 m (± 71 m) to 6 m (±56 m). Both horizontal and vertical precisions improved after deployment. Horizontal precision was 7.10 m (± 23 m) before and 6.72 m (± 19.7 m) after deployment (χ2 = 4543.2, df = 1, p-value <0.001), and vertical precision improved from 11m (± 85 m) to 10 m (± 67 m) after deployment (χ2 = 6824.0, df = 1, p-value <0.001) (Table 1).

## Discussion

In this study, we quantify the accuracy and precision of Flyway 50 Movetech Telemetry tracking devices and assess its suitability for studies that require high spatial resolution. Horizontal (3.40 m at 1 min fix interval) and vertical (4.95 m at 1 min fix interval) accuracies improved with decreasing fix intervals and were not negatively affected after deployment on birds. This accurate spatial resolution data enables ecological and behavioral studies that require highly accurate and precise information.

### GPS fix interval

The fix interval influenced the accuracy and precision of the devices, with a loss of 3.10 m and 4.74 m in horizontal and vertical accuracy, respectively, from the 1 min to 60 min interval (6.50 m horizontal and 9.69 m vertical accuracy at 60 min fix interval). While we found significant variability in device performance, all except one device, had lower accuracy in the 20 min and 60 min fix intervals compared to the device programmed with 1 min fix intervals. Thus, these results support previous findings that longer fix intervals have a negative effect on location accuracy [9, 24, 49]. The GPS units store information on the satellite constellation of the previous fix for a period of time (ephemeris retention), which increases the performance of the device when calculating a new location [9], by increasing GPS location acquisition success [6] and providing a fix in a shorter period of time [14], usually designated as a warm start. However, Cain III et al. [6] did not find an effect of fix interval on accuracy and Jiang et al. [14] found that the positions obtained with longer fix intervals (60 min) had lower DOP than in shorter intervals. Forin-Wiart et al. [10] found higher location errors in the 5 min interval, compared to 15 and 60 min intervals. They proposed that given the high temporal correlation between fixes, a low accuracy location would influence the following GPS position, decreasing the overall accuracy of the device. Our findings do not support this theory, even with similar fix intervals and in similar, open area habitat. This difference in results highlights the importance of testing the GPS units from different manufacturers, as they might produce different results [6, 15, 30]. Moreover, with newly developed loggers that collect data in different intervals according to the battery performance (e.g. dynamic fix transmitters [17]), the fix interval should be taken into consideration when accounting for device accuracy.

### Performance after deployment

After deployment on white storks, we did not find a decrease in horizontal accuracy. In fact, the devices performed slightly better after deployment than before (increase in 1 m vertical accuracy and 0.38 m in horizontal precision and 1 m in vertical precision).

The performance of GPS devices can be influenced by environmental factors, such as topography [6] and habitat [7, 23]. Vegetation structure and proximity to buildings might also decrease the sky availability and reflect the GPS signal, which increases location error [23]. In our study design, we avoided topography and habitat bias by performing the stationary tests geographically close to the deployment locations. However, the stationary test before deployment was performed with our tested devices in close proximity to each other (less than 2cm apart) and on the cement structure the triangulation station, which could have decreased the accuracy of the devices before deployment due to the reflection of the GPS signal.

**Fig 5:**
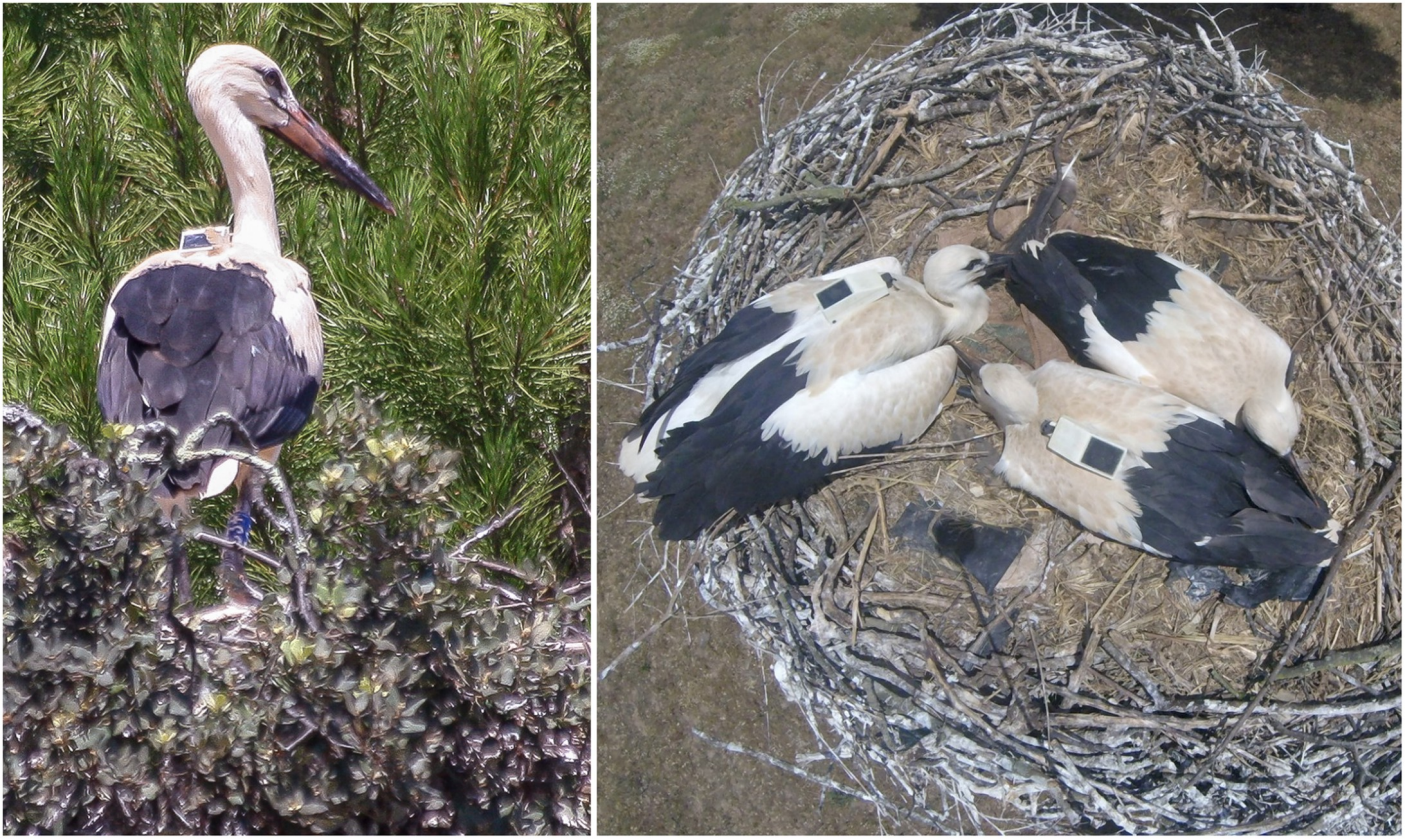
Position of the tracking devices on the back of pre-fledging white storks.

Moreover, after deployment, the storks were on nests located on top of high trees with uninterrupted view of the sky, which could have slightly increased the accuracy and precision of the devices. Other studies have found that animal movement [7, 16, 26], behavior [25, 27] morphology of the animal [26] and tag attachment method [50] can restrain the signal reception. The angle of the GPS antenna in relation to the sky has been found to influence the performance of the device, with lower fix acquisition success [8, 28] and lower accuracy [10, 22, 51] when the antenna is not directly facing the sky. When estimating the post-deployment device accuracy and precision, we prevented animal movement and behavior bias by deploying the devices on birds before fledging. However, device position varied between 0°, when the bird is lying on the nest, and close to 80° when the bird is standing (Fig 5). Despite this large variation in antenna position, white stork chicks spend a large proportion of the time lying on the nest (pers. obs.), therefore the influence of GPS antenna position on device performance was likely negligible.

Finally, although GPS signal travels through leaves, tree trunks and animal’s bodies, there is a reduction of the signal strength and the degree of attenuation depends on the material and distance that the signal has to travel [52]. Our deployed devices were tested within a reinforced housing, with thicker nylon (between 3–4 mm thickness), which might have increased the number of low accuracy positions when compared to the initial stationary test (Table 1). Nevertheless, after deployment the tested devices proved to be highly accurate, with over 84% and 71% GPS positions with less than 10 m horizontal and vertical error, respectively.

### Elimination of inaccurate positions

Despite slight losses of accuracy with fix interval, the tested devices were still highly accurate. Combining all fix intervals during the stationary test, 95% of the positions were within 11 and 18 m horizontal and vertical error, respectively. Montgomery et al. [31] found that in small-scale ecological studies (<5 ha size patch), using a 10 m resolution categorical raster, a mean GPS accuracy of <5 m was needed to obtain 90% accurate inferences. An accuracy of <5m could be obtained with the tested devices tested if the fix interval was set between 1 and 20 min intervals. Using such high frequency fix intervals can thus help identify species’ fine-scale movement patterns and habitat requirements, critical for designing suitable conservation actions in local scales, though it can also compromise the device’s battery life and the duration of the study [53].

Despite the high accuracy of the devices, there was a small number of locations with errors above 250 m, both horizontally and vertically. These highly inaccurate positions can lead to a decrease in performance of habitat selection models [32]. For studies requiring very highly accurate GPS locations, such as studies in fragmented landscapes (e.g. urban areas, [23]), or studies of collision with human infrastructures (e.g. wind-farms [37, 38]), it is important to be able to identify and eliminate outlier positions to increase GPS accuracy.

The most commonly used metrics to filter large error in GPS positions are the number of satellites [5, 13, 21] and DOP [13, 14, 18, 19, 22, 23]. However, these can result in the elimination of a large proportion of the dataset, including accurate positions, while not eliminating all inaccurate positions [13, 16, 21, 54]. Estimating the true altitude error and relate it to the horizontal error, produces acceptable results in eliminating poor quality fixes in comparison with single metric models [55]. This method however is only suitable for broad-scale habitat analysis, and since it relies on knowing the exact altitude of the animal, it is not appropriate for arboreal or flying species.

The devices tested in this study provide a *GPS-Error* estimate that proved to be effective at identifying low accuracy positions in short fix intervals (1 min), but it was not possible to replicate the results with longer fix intervals. Moreover, since the performance of the device is related to the habitat, by excluding locations with large positional errors there can be a bias in excluding data related to a single habitat [13, 21, 29, 30, 33, 55]. Using species-specific GPS metadata, such as unrealistic speed, turning angles or distances travelled between consecutive fixes is effective in eliminating large positional errors [54]. However, this method is dependent on the mobility of the species, as well as the fix interval [55]. Other modelling techniques, such as using sensors (accelerometers and magnetometers) and GPS drift-corrected dead reckoning, have successfully increased the accuracy of animal movement estimates in low intensity GPS schedules [56]. This is particularly important in non-solar tags in which, in order to maximize the lifespan of the battery, longer fix intervals are used.

## Supporting information

S1 File (XLSX)
